# Evaluation of Olfactory Function by Iranian Smell Diagnostic Test in Patients with Parkinson's disease in North of Iran

**DOI:** 10.22038/ijorl.2021.50564.2688

**Published:** 2021-09

**Authors:** Keyvan Kiakojuri, Leila Pouladi, Payam Saadat, Alijan Ahmadi Ahangar, Hemmat Gholinia

**Affiliations:** 1 *Clinical Research Development Center, Rohani Hospital, Babol University of Medical Sciences, Babol, Iran.*; 2 *Department of Obstetrics and Gynecology, Babol University of Medical Sciences, Babol, Iran.*; 3 *Mobility Impairment Research Center, Health Research Institute, Babol University of Medical Sciences, Babol, Iran.*

**Keywords:** Olfactory dysfunction, Olfactory, Parkinson's disease, Severity of Parkinson's disease

## Abstract

**Introduction:**

Parkinson's disease is a neurodegenerative and multisystem disorder affecting systems more than the motor system. The olfactory disorder is an early non-motor symptom of Parkinson's disease.

**Materials and Methods:**

The present study was conducted on 110 patients aged 50-95 years with a diagnosis of Parkinson's disease referred to the Neurology Clinic of Babol University of Medical Sciences between 2018-2019. The control group consisted of 50-95-year-old non-neurological patients who were matched for age and gender with patients with Parkinson's disease. Data were collected by examination, demographic and clinical information questionnaire (duration of disease, the severity of disease, symptom index), as well as Iranian smell diagnostic test. A p-value less than 0.05 was considered statistically significant.

**Results:**

The mean age scores of Parkinson's disease and control groups were obtained at 69±9 and 66±9 years, respectively. The mean duration of the disease was 5 years. Patients with Parkinson's disease scored lower on the Iranian smell test, and olfactory function was significantly reduced in the case group (P<0.001). Based on the results, olfactory function in patients with Parkinson's disease was not significantly correlated with gender, marital status, education, place of residence, and occupation(P<0.05). Only olfactory dysfunction was increased with age (P=0.01). In addition, olfactory dysfunction showed no significant relationship with severity of disease, duration of disease, and clinical index sign. Rapid Iranian smell test with a cut-off of 3.5% had a sensitivity of 87.3% and a specificity of 66.4%.

**Conclusion:**

According to the obtained results, olfactory dysfunction is an important non-motor and a primary symptom in patients with Parkinson's disease and is not related to the duration and severity of motor symptoms and symptom index.

## Introduction

Parkinson's disease is one of the most common motor disorders and the second most commonly reported neurodegenerative disease of the central nervous system (1). Nowadays, 1%-2% of the population over the age of 65 years suffer from Parkinson's disease, and this figure rises to 3%-5% in people aged 85 years and over (2). Parkinson's disease is a multisystem disorder which affects other systems more than the motor system (3). Olfactory disorders are a dominant non-motor symptom in patients with Parkinson's disease. The prevalence of these disorders has been reported as 45-49% and 90% in the studies conducted by Ansari et al. and Doty et al., respectively (4-5). Decreased olfactory sensitivity predominates motor symptoms in Parkinson’s disease. The motor symptoms do not appear until the destruction of 80% of dopaminergic neurons in the substantia nigra (6). Therefore, early detection is of utmost importance for effective intervention in Parkinson's disease. It is hypothesized that olfactory disorders are not merely a simple primary symptom and are more likely to be a major culprit behind Parkinson's disease. Some investigations reported that decreased olfactory sensitivity in Parkinson's disease is largely independent of disease duration, considering the severity of motor symptoms. Nevertheless, there is little evidence of its relationship with disease duration or severity (5,7). This discrepancy may be ascribed to different measurement methods of the olfactory disorder (8). Some studies showed a decrease in selective olfactory sensitivity (9). When the sample population is limited, it may not be possible to elucidate the relationship of decreased olfactory function with disease severity and duration (10). Despite the high prevalence of Parkinson's disease in the elderly and the need for early diagnosis of this disease, no standardized study has been conducted on patients with Parkinson's disease in Iran, and available studies are filled with serious inconsistencies. Therefore, this case-control study was carried out on two groups, including patients with Parkinson's disease referred to the neurology clinic as the case group and healthy controls without any risk factor for decreased odor sensitivity referred to non-neurological clinics as the control group. The present study investigated the reduction in olfactory sensitivity and its association with demographic information, stage of disease, the severity of motor symptoms, and duration of Parkinson's disease.

## Materials and Methods

**Study population**

This case-control study which was approved by the Ethics Committee of Babol University of Medical Sciences was conducted on 110 patients with Parkinson's disease aged 50-95 years referred to a neurology clinic during 2018-201. The control group included 75 subjects without Parkinson's disease aged 50-95 years referred to other clinics of Ayatollah Rouhani Hospital in Babol. The participants in the case and control groups were matched for age and gender. Before the commencement of the study, the subjects with risk factors for a decreased sense of smell, including diseases of the nose and paranasal sinuses, such as polyps, rhinitis, rhinosinusitis, rhinoplasty, or other reconstructive interventions, were excluded. The inclusion criteria entailed idiopathic Parkinson's disease diagnosis based on common criteria and four classic symptoms and patient satisfaction for performing the olfactory diagnostic test. On the other hand, the exclusion criteria were as follows: 1) secondary Parkinson's, 2) Parkinsonism, 3) Parkinson's Plus, 4) a risk factor for olfactory dysfunction (including a history of trauma to the head, nasal diseases, and diseases of paranasal sinuses, such as polyp, rhinitis, rhinosinusitis, nasal cosmetic surgery, as well as rhinoplasty and other interventions), 5) brain neoplasms, 6) smoking, 7) other neurodegenerative diseases, such as Alzheimer's, ***multiple sclerosis***, and epilepsy, 8) mental disorders, such as schizophrenia, depression, 9) recent common colds, 10) migraines, 11) diabetes, 12) pregnancy, 13) long exposure to chemicals, and 14) a history of the olfactory disorder. After obtaining written consent from patients to participate in the study, demographic information, including age, gender, education, occupation, marital status, place of residence of both groups, and duration of Parkinson's disease, was included in the checklist. In the Parkinson's and control groups, the subjects with an impaired sense of smell were assessed by an otolaryngologist and excluded, and a neurologist was assigned to determine the severity of the disease and the priority of the four symptoms. The severity of Parkinson's disease was determined based on the Unified Parkinson's Disease Rating Scale (UPDRS) which is presented by the Motor Disorders Society (MDS) and is based on the modified Hoehn and Yahr Scale. In the control group, a complete examination was carried out by a neurologist who planned to rule out Parkinson's disease. 

**Assessment of olfactory function**

The case and control groups were assessed by the Iranian diagnostic olfactory test. The Iranian olfactory diagnostic test that is the localized version of the University of Pennsylvania Smell Identification Test (UPSIT) assesses olfactory function in the Iranian population. Iran SIT was adopted in 2015 by Taherkhani et al. (15) Firstly, familiar smells for the Iranian population were selected by the students from all over the country present in the dormitories of Tehran. The odors were then matched to UPSIT. Finally, out of 40 selected odors, 24 odors were approved; accordingly, the diagnosis of normosmia, mild hyposmia, severe hyposmia, and anosmia was evaluated for the Iranian population (15). The present study used a rapid olfactory test with six tests for evaluation. The case and control patients in each test selected the correct option from the four available odors which can be scratched by paper.

**Statistical analysis**

The data were analyzed in SPSS software (version 22) using chi-square, t-test, Pearson correlation coefficient, logistic regression analysis, and Rock Curve. A p-value less than 0.05 was considered statistically significant.

analysis, and Rock Curve. A p-value less than 0.05 was considered statistically significant.

## Results

This case-control study was conducted on 110 patients aged between 50-95 years (mean age of 69±9 years), and 110 controls (50-95 years old) with a mean age of 66±9 years. Table 1 displays the demographic characteristics of the participants. Regarding occupation, in the Parkinson group, 14 (12.7%) patients were retired employees, 49 (44.5%) cases were freelancers, and 47 (42.7%) subjects were unemployed. In the control group, 21 (19.1%) cases were retired employees, 31 (28.2%) participants were freelancers, and 58 (52.7%) subjects were unemployed. In terms of occupation and Parkinson's disease, more Parkinson's patients were self-employed than controls, and this relationship showed a significant relationship. (P=0.03). There was no significant difference between the two groups of Parkinson's patients and the control group regarding other demographic characteristics, including gender (P=0.17), age (P=0.22), marital status (P=0.11), level of education (P=0.39), and place of residence (P=0.16).Table 2 depicts the frequency distribution of clinical features in Parkinson's patients. The mean duration of illness was obtained at 5.5±4.85 years. The majority of patients (36.4%) were in the age range of 5-9 years. Most of the patients (44.5%) were in stages 2.5-3, and mean disease severity was reported as 3.88±1.36. The most prominent and dominant clinical symptom among the four symptoms of Parkinson's patients was related to tremor, and the least index symptom was related to gait and postural disorders.

**Table 1:** Frequency distribution of demographic characteristics in Parkinson and control groups (Chi-square test)

**Variables**		**Patients (%)** **(n=110)**	**Controls (%) (n=110)**	**P-value**
Gender	Female	64 (58.2)	54(49.1)	0.17
	Male	46 (41.8)	56(50.9)	
Age (year)	50-64	34 (30.9)	46(41.8)	0.22
	65-79	61(55.5)	53(48.2)	
	80-95	15 (13.6)	11(10.0)	
Marital status	Single	6 (5.5)	1(0.9)	0.11
	Married	91 (82.7)	99(90.0)	
	Divorced	13 (11.8)	10(9.1)	
Education	Illiterate	71 (64.5)	66(60.0)	0.39
	High school	31(28.2)	30(27.3)	
	Diploma and higher	8 (7.3)	14(12.7)	
occupation	Retired employee	14 (12.7)	21(19.1)	0.03
	Freelancer	49 (44.5)	31(28.2)	
	Unemployed	47 (42.7)	58(52.7)	
Place of residence	Urban	62(56.4)	72(65.5)	0.16
	Rural	48(43.6)	38(34.5)	

**Table 2:** Frequency distribution of clinical features in patients with Parkinson's disease

**Variables**		**Number**	**Percent**
Duration of illness	One year and less	22	20.0
	More than one -4 years	30	27.3
	5-9 years	40	36.4
	More than 9 years	18	16.3
Disease severity	1-2	47	42.7
	2.5-3	49	44.5
	4-5	14	12.7
Index symptoms	tremor	73	66.4
	Rigidity	27	24.5
	Bradykinesia	6	5.5
	Gate disorder	4	3.6

Table 2 depicts the frequency distribution of clinical features in Parkinson's patients. The mean duration of illness was obtained at 5.5±4.85 years. The majority of patients (36.4%) were in the age range of 5-9 years. Most of the patients (44.5%) were in stages 2.5-3, and mean disease severity was reported as 3.88±1.36. The most prominent and dominant clinical symptom among the four symptoms of Parkinson's patients was related to tremor, and the least index symptom was related to gait and postural disorders. As demonstrated in Table 3, based on olfactory function, Parkinson's and control groups were divided into three groups of no-smell (anosmia): 0-1, the olfactory decline (hyposmia): 2-4, and normal olfactory (normosmia): 5-6. The majority of subjects with Parkinson's disease were in the hyposmia group (olfactory deficiency), while most of the control group were in the normosmia group. Furthermore, there was a significant relationship on olfactory dysfunction in both Parkinson's and control groups, and olfactory function significantly decreased in the patients with Parkinson's disease (P<0.001). Regarding olfactory function in patients with Parkinson's disease and its relationship with demographic indices as displayed in Table 4, males outnumbered females in the anosmia group, and females outnumbered males in the normosmia group, and this relationship was statistically significant (P= 0.05). In terms of olfactory function in patients with Parkinson's disease and age, it can be stated that the highest number of hyposmia was observed in all age groups; however, the number of normosmia decreased significantly with age (P= 0.01). Marital status, educational level, occupation, and place of residence in patients with Parkinson's disease were not correlated with olfactory function. 

**Table 3:** Classification of olfactory function in patients with Parkinson's disease and control group (Chi-square test)

**Olfactory function**	**Parkinson’s disease (%)**	**Control (%)**	**P-value**
Anosmia	34 (30.9)	0(0)	P<0.001
Hyposmia	61 (55.5)	35(31.8)	
Normosmia	15 (13.6)	75(68.2)	

**Table 4:** Relationship between olfactory function and demographic parameters in Parkinson's patients(chi-square test)

**Variables**		**Olfactory function**			**P-value**
		**Anosmia**	**Hyposmia**	**Normosmia**	
Gender	Male	(24(37.5	35(54.7)	5(7.8)	0.05
	female	10(21.7)	26(56.5)	10(21.7)	
Age (year)	50-64	4(11.8)	21(61.8)	9(26.5)	0.01
	65-79	24(39.3)	31(50.8)	6(9.8)	
	80-95	6(40.0)	9(60.0)	0(0.0)	
Marital status	Single	1(16.7)	4(66.7)	1(16.7)	0.33
	Married	26(28.6)	53(58.2)	12(13.2)	
	Divorced	7(53.8)	4(30.8)	2(15.4)	
Education	Illiterate	25(35.2)	37(52.1)	9(12.7)	0.28
	High school	8(25.8)	17(54.8)	6(19.4)	
	Diploma and higher	1(12.5)	7(87.5)	0(0.0)	
Occupation	Employee	5(35.7)	8(57.1)	1(7.1)	0.12
	Freelance	18(36.7)	28(57.1)	3(6.1)	
	Unemployed	11(23.4)	25(53.2)	11(23.4)	
Place of residence	Urban	(18(37.5)	26(54.2)	4(8.3)	0.22
	Rural	16(25.8)	35(56.5)	11(7.17)	

As illustrated in Table 5, olfactory function is not significantly correlated with disease duration, disease severity, and clinical index (r=-0.083 and P=0.39, r=-0.11, P= 0.21, P=0.82 respectively). 

**Table 5:** Relationship between olfactory function and clinical indicators in patients with Parkinson's disease (chi-square test)

**Variable**		**Olfactory function**			**P-value**
		**Anosmia**	**Hyposmia**	**Normosmia**	
Duration of illness	One year and less	6(27.3)	13(59.1)	3(13.6)	0.39
	2-4	12(40.0)	13(43.3)	5(16.7)	
	5-9	8(20.0)	26(65.0)	6(15.0)	
	More than nine	8(44.4)	9(50.0)	1(5.6)	
Disease severity	0-2	12(25.5)	28(59.6)	7(14.9)	0.21
	2.5-3	14(28.6)	29(59.2)	6(12.2)	
	More than three	8(57.1)	4(28.6)	2(14.3)	
Index symptoms	tremor	23(31.5)	39(53.4)	11(15.1)	0.82
	rigidity	3(50.0)	3(50.0)	0(0.0)	
	bradykinesia	7(25.9)	16(59.3)	4(14.8)	
	Gate disorder	1(25.0)	(3(75.0	0(0.0)	

**Fig 1:**
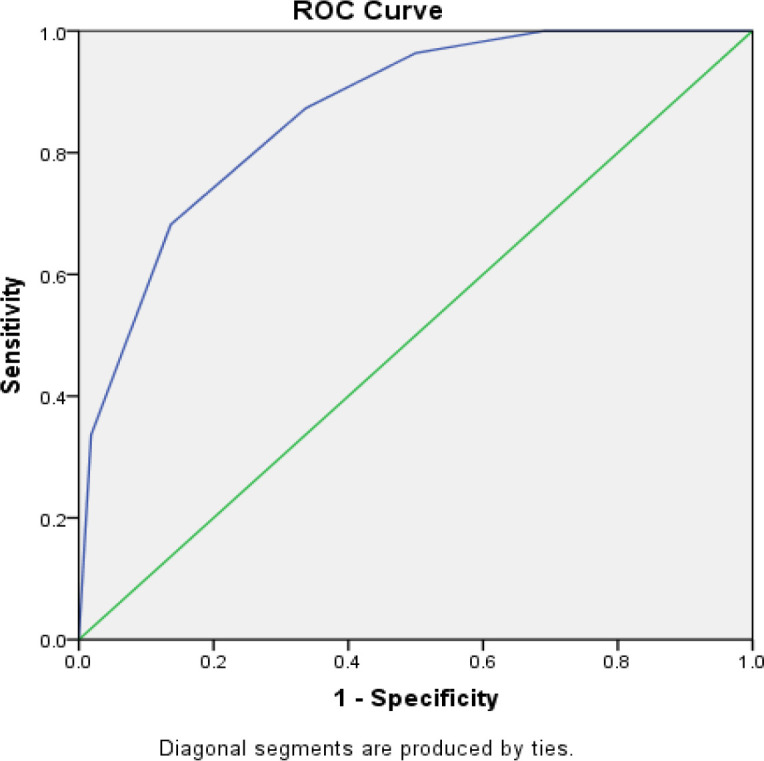
Receiver operating characteristic curve of Iran-SIT rapid test

According to Table 6, in the Parkinson's disease group, the highest odor detected by patients was a mint odor, and the lowest odor was reported in rose water and cologne. In the control group, the highest detected odor was a mint odor, while the lowest odor was related to cologne. There was a significant difference in the diagnosis of all six odors evaluated in the Iran-SIT test between patients with Parkinson's disease and the control group (P<0.001). All the evaluated odors were detected by more than 70% of subjects in the control group. As depicted in Figure 1, the cut-off value for the olfactory test score was considered to be 3.5 with a sensitivity of 87.3% and a specificity of 66.4%.

**Table 6:** Evaluation of rapid Iranian olfactory Test in patients with Parkinson's disease and healthy controls (chi-square test)

**Olfactory function test**		**Parkinson (%)**	**Control (%)**	**p-value**
Smell of bananas	True	46 (41.8)	92 (83.6)	P<0.001
	False	64 (58.2)	18 (16.4)	
Smell of rose water	True	40 (36.4)	83 (75.5)	P<0.001
	False	70 (63.6)	27 (24.5)	
Smell of cinnamon	True	44 (40.0)	86 (78.2)	P<0.001
	False	66 (60.0)	24 (21.8)	
Smell of garlic	True	42 (38.2)	94 (85.5)	P<0.001
	False	68 (61.8)	16 (14.5)	
Smell of mint	True	62 (56.4)	98 (89.1)	P<0.001
	False	48 (43.6)	12 (10.9)	
Smell of cologne	True	40 (36.4)	81(73.6)	P<0.001
	False	70 (63.6)	29 (26.4) c	

## Discussion

This case-control study aimed to assess olfactory function in patients with Parkinson's disease by Iranian smell diagnostic test in a tertiary hospital in the north of Iran. The obtained results demonstrated that there was a significant relationship on olfactory dysfunction in both Parkinson's and control groups, and olfactory function significantly decreased in patients with Parkinson's disease. Gender, age, marital status, education, and place of residence in patients with Parkinson's disease and control group were evaluated, and no significant relationship was reported. Only in the variable of occupation, there was a significant relationship in patients with Parkinson's disease, compared to the control group, and in the Parkinson's group, the number of freelancers was higher (P<0.001). 

The literature search yielded no finding on the relationship of olfactory dysfunction with marital status, occupation, and place of residence in Parkinson's patients. Nevertheless, the present research differed from these studies in the type of selection and the number of participants. Doty et al. conducted a study in 1995 on 180 patients with Parkinson's disease and 612 controls. Most of the patients were in the first stage of the disease and were evaluated by the UPSIT test. The findings of the mentioned study indicated that in patients with Parkinson's disease, men and older patients obtained lower scores in Pennsylvania olfactory test (11). 

In the present study, no significant relationship was detected between olfactory function and gender in Parkinson's and control groups (P>0.05). In line with the referred study, there was a significant relationship between olfactory function and age in patients with Parkinson's disease, and patients in the higher age group had lower performance (P<0.01). This association with age in both studies can be ascribed to the effect of aging on the olfactory decline. Cavaco et al. (2015) assessed the association between olfactory dysfunction and Parkinson's disease severity in a case study of 166 nondemented patients with Parkinson's disease diagnosis (according to the United Kingdom Brain Bank criteria) from Centro Hospital do Porto's movement disorders outpatient clinic, compared to 388 healthy participants. 

Based on the Brief Smell Identification Test (B-SIT) score, the prevalence of abnormal olfaction in Parkinson's disease was 82%, while no significant associations were found with gender, age, education, disease, and olfactory dysfunction durations scores (16). In a similar vein, in the current study, olfactory dysfunction showed no significant associations with gender, occupation, education, and the place of residence. This similarity is due to the fact that olfactory decline may be related to the mechanism of Parkinson's disease and has little to do with other factors. However, in the present study, age had a significant negative relationship with the olfactory function of Parkinson's disease (i.e., their olfactory function deteriorated with an increase in age).

In a study by Cavaco et al., 82% of Parkinson's disease patients had olfactory dysfunction ( i.e., patients with abnormal B-SIT scores showed more PD severity than Parkinson's disease patients with normal B-SIT scores).

 In other words, patients with abnormal B-SIT scores had higher Hoehn and Yahr stage (P=0.006) and more severity in UPDRS-III (sub-scale 3) (P=0/018). However, as mentioned earlier, the duration of the disease was not a good predictor. In our study, the duration of the disease is consistent with the present study, but the severity of the disease is not directly related to increased olfactory dysfunction and is not consistent with our study (16).

In the study by Cavaco et al., although not statistically significant, the frequency of abnormal olfactory dysfunction tended to be lower among patients with tremor-dominant phenotype, as compared to that in those with PIGD subtype. This finding is in agreement with the results of a study performed by Stern et al. (17).

 In addition, another study by Iijima et al. investigated the olfactory function using an odor stick identification test for Japanese (OSIT-J) on 54 patients with idiopathic Parkinson’s disease. The results of the stated research indicated that the number of correct responses in patients with Parkinson's disease was significantly lower, compared to those reported in the control group. Furthermore, the olfactory test score was not correlated with gender, disease severity, disease duration, or medication (12). 

In the present study, disease duration, disease severity, and the symptom of the disease index had no significant relationship with olfactory function. Iijima et al. (2011) also noted that Subjective symptoms of olfactory dysfunction were significantly higher (P<0.05) in the akinetic-rigid type (ART), as compared to those in the tremor‐dominant type (TDT) (18). Furthermore, in the present study, patients with remarkable symptoms of tremor‐dominance consisted the majority of the normosmia group, while no patient with ART was found in the normosmia group; however, no statistically significant relationship was reported in this regard Another study using 40UPSIT test and Sniffin' Sticks test-16 (​SS-16) in the diagnosis of Parkinson's disease in Brazil reported the specificity of 83.5% and the sensitivity of 82.1% for 40 UPSIT test, as well as 89% specificity and 81.1% sensitivity for 16SS. The results of the mentioned study point to the usefulness of 40UPSIT and 16SS in early diagnose of Parkinson's disease (13). In the present study performed with the rapid Iranian olfactory test, sensitivity and specificity were obtained at 87.3% and 66.4%, respectively (cut-off=3.5). In the same vein, in previous studies, olfactory dysfunction demonstrated no significant relationship with gender, age, educational level, and disease duration. Moreover, it has been reported that olfactory dysfunction due to Parkinson's disease cannot be influenced by demographic variables. In the same way, the findings of the current study indicated no significant relationship between olfactory function and gender, occupation, education, and place of residence in patients with Parkinson's disease. Nonetheless, only age had a reverse significant relationship with olfactory function, and olfactory function was decreased with age. On the contrary, in the study by Seena vengalil et al., no significant relationship was found between the age of Parkinson's disease patients and the Pennsylvania olfactory test score. In addition, consistent with the present study, olfactory function showed no significant relationship with the olfactory test score, disease severity, and disease duration (14).

In a study by Vengalil et al., apple was the most discriminant odor found. The Parkinson and control groups significantly differed in the discrimination of 24 out of 40 odors. It was attributed to the congenital hyposmia or acquired loss of olfactory cells due to exposure to a neurotoxic vapor (14). In the present study, the most discriminant odor found by the patients was mint to which 62 patients (56/4%) gave correct responses. On the other hand, the least discriminant odors were perfume and rose-flowered liquor to which 70 (63/6 %) cases gave incorrect responses. Within the control group, mint was found to be the most discriminant odor with 95 (89/1%) correct responses, and the least discriminant odor was perfume to which 92 people (26/4 %) gave incorrect responses. In general, a statistically significant difference was found between the Parkinson's disease patients and the control group in discriminating all of the six odors in Iran-SIT (P<0/01). All of the evaluated odors were discriminated correctly by more than 70% of cases in the control group. 

Picillo et al. developed a culturally adapted version of the UPSIT-40 to compare the performance of 61 nondemented Italian controls with 68 Parkinson's disease patients. The Parkinson's disease group gained significantly lower scores (16.8±4.9), as compared to the control group (26.6±5.7) (P<0.001). The UPSIT-40 score differentiated Parkinson's disease and control subjects, and more than 50% of control group participants responded correctly to 30 out of the total 40 odors. Therefore, this adapted version of UPSIT-40 was used as a useful diagnostic tool for accuracy improvement of Parkinson's disease diagnosis in the Italian population, alongside the clinical examination. Moreover, 10 out of 40 odors were discriminated by less than 50% of the Italian subjects in the control group, signifying the inappropriateness of these odors for the Italian population. Finally, 28 out of 40 odors were selected as the main discriminating ones between the Parkinson's disease and the control group.

In the present study, the Iran-SIT showed a significant difference between Parkinson's disease and the control groups in all of the six used odors (P=0.01). In addition, the results of the study by Picillo et al. demonstrated that the duration of the disease (P=0.01) and Hoehn and Yahr criteria (P=0.01) were not correlated with an increase in olfactory dysfunction. The Italian version of the UPSIT-40 showed a sensitivity of 82 % and a specificity of 88.2 % (19). The present study is in agreement with the research by Picillo in the duration and severity of the disease; however, in the present study, all of the odors were discriminated by 70% of the control group; therefore, they were deemed valid for the Iranian population. Tissingh et al. (2001) studied 41 nondemented Parkinson's disease patients, out of whom 24 cases had untreated early Parkinson's disease, and 18 subjects were included in the control group). 

Odor identification and discrimination data were used for odor detection scores. Parkinson's disease patients gained significantly lower scores on all olfactory tests, compared to healthy subjects. Moreover, in comparison with the healthy subjects, the subgroup of de novo patients with early Parkinson's disease also showed significant olfactory disturbances. Furthermore, in contrast with the finding of the current study, odor discrimination measures were related to disease severity (9).

**Strengths and limitations**

Among the notable strengths of the present study, we can refer to the assessment of reduced olfactory sensitivity and its relationship with demographic information, disease stage, the severity of motor symptoms, and Parkinson's disease duration. It was not possible to use the olfactory test of 24-odor. Also, the lack of cooperation of patients to go to a public clinic to adjust the dose of the drug is considered a limitation.

## Conclusion

As evidenced by the obtained results, the Iranian olfactory test and its dysfunction can be used as an efficient tool for the detection of one of the early symptoms of Parkinson's disease.
